# Effect of CPAP on blood glucose fluctuation in patients with type 2 diabetes mellitus and obstructive sleep apnea

**DOI:** 10.1007/s11325-021-02556-0

**Published:** 2022-02-15

**Authors:** Xin Zhao, Wei Zhang, Sixu Xin, Xiaofeng Yu, Xiaomei Zhang

**Affiliations:** 1 Department of Endocrinology, Peking University International Hospital, No. 1, Life Garden Road, Zhongguancun Life Science Garden, Changping District, Beijing, 100001 China; 2 Sleep Center Department, Peking University International Hospital, Beijing, 100001 China

**Keywords:** Obstructive sleep apnea, Type 2 diabetes mellitus, Continuous glucose monitoring system, Continuous positive airway pressure

## Abstract

**Purpose:**

This study aimed to investigate the effect of continuous positive airway pressure (CPAP) on blood glucose fluctuation in patients with type 2 diabetes mellitus (T2DM) and obstructive sleep apnea (OSA).

**Methods:**

Patients with T2DM and OSA were divided into an intervention group and a control group. All patients were treatment naïve. The intervention group was given CPAP therapy. The subjects were monitored using a continuous glucose monitoring system (CGMS) for 2 weeks.

**Results:**

Of 60 patients, 30 were selected to receive CPAP intervention while 30 without CPAP served as controls. The CPAP tolerance of the intervention group was good, with average time on CPAP therapy of 55.2 ± 4.3 days, and average daily time on CPAP therapy of 8.3 ± 2.8 h. The postprandial blood glucose (PBG), fasting blood glucose (FBG), and HbA1c levels in the intervention group decreased significantly (*P* < 0.05). Significant variations in 24-h mean blood glucose and night-time mean blood glucose were significantly lower with CPAP therapy than without therapy (*P* < 0.05, respectively). The mean of daily differences and mean ambulatory glucose excursions were both considerably lower with treatment than without (*P* < 0.05, respectively). There was also a significant difference in time in range and time above range (*P* < 0.05, respectively).

**Conclusion:**

CPAP treatment may significantly improve the blood glucose level and blood glucose stability in patients with T2DM and OSA. CPAP is an effective treatment method beyond lifestyle intervention and drug therapy.

## Introduction

Because type 2 diabetes mellitus (T2DM) and obstructive sleep apnea (OSA) are both highly prevalent diseases, they frequently occur together. Furthermore, patients with T2DM have a considerably greater prevalence of OSA than the overall population. The prevalence of OSA in patients with T2DM is about 55% (24–86%), and the risk is 50% higher than that of patients without T2DM [1–3]. OSA affects more than 60% of hospitalized patients with T2DM. Studies have recently shown that patients with T2DM and OSA have a significantly higher risk of macrovascular and microvascular complications [4, 5]. Intermittent hypoxia produced by nocturnal apneas or hypoventilation during sleep may cause blood glucose fluctuations and impair insulin sensitivity.

CPAP is the treatment of first choice for patients with OSA [6]. As CPAP improves nocturnal hypoxia in patients with OSA, it may also improve the metabolic disorders in those patients.

With the advancement of continuous glucose monitoring systems (CGMS) in recent years, researchers have reported that blood glucose fluctuations, rather than glycosylated hemoglobin (HbA1c), play a significant role in the complications of diabetes. Blood glucose fluctuations have been linked to complications such as coronary microangiopathy, diabetic retinopathy, and diabetic nephropathy [7, 8].

Therefore, the goal of this study was to assess how blood glucose levels and fluctuations in glucose change in patients with T2DM and OSA before and during CPAP treatment.

## Methods

### Research subjects

Patients with T2DM patients and snoring symptoms in the endocrinology department of the International Hospital of Peking University were evaluated by polysomnography (PSG) and a sample of patients with OSA were enrolled from December 2019 to June 2021. All patients were naive to CPAP and had no prior surgery for OSA. Diet, lifestyle, and hypoglycemic drugs did not change before and during the study. The 1999 WHO diagnostic criteria were used to confirm diagnosis of T2DM: (1) fasting plasma glucose (FPG) ≥ 7.0 mmol/L. (2) Postprandial blood glucose was ≥ 11.1 mmol/l at 2 h or ≥ 11.1 mmol/l randomly. In the absence of clear hyperglycemia syndrome, the standard was confirmed by repeated testing. Criteria for exclusion were: (1) sinusitis, nasal polyps, deviation of the nasal septum, tongue hypertrophy, tonsil hypertrophy, hyperplasia of lymph tissue in the root of the tongue, and other anatomical stenosis of the respiratory tract; (2) endocrine diseases such as hypothyroidism, acromegaly, and adrenal hyperplasia; (3) patients who took insulin-sensitive drugs. The study was approved by the Peking University International Hospital’s Bioethics Committee. Participants gave written informed consent. The registry’s URL is www.chictr.org.cn and the trial’s registration number is ChiCTR1900023467.

### General conditions

Medical records were obtained for all subjects in this study. Medical history and important clinical markers were documented including gender, age, weight, height, hip, waist, diabetes duration, diastolic blood pressure (DBP), and systolic blood pressure (SBP). Waist-to-hip ratio (WHR) was calculated by the formula waist/hip, and the formula weight/height^2^ (kg/m^2^) was used to compute the body mass index (BMI).

### Polysomnography monitoring

Subjects were monitored in the sleep monitoring center in Peking University International Hospital for at least 7 h at night. The instrument was an Alice A6 (Philips, USA) multi-channel sleep monitor, which recorded (1) mouth and nose airflow; (2) snoring; (3) mean SaO_2_ and the lowest SaO_2_; (4) chest and abdominal breathing movement. The apnea hypopnea index (AHI) was established by international standards of the American Academy of Sleep Medicine (AASM) Sleep Apnea Definitions Task Force [9] as the average number of apneas and hypopneas during at least 7 h of sleep. Patients with an AHI ≥ 5 and mostly obstructive events were diagnosed with OSA. Patients were classified into two groups based on the results of the PSG: severe (AHI ≥ 30 times/h) and mild to moderate (AHI 5 to < 30 times/h). On the day of the monitoring, patients were advised not to take sedatives, strong tea, wine, or caffeine.

### Continuous glucose-monitoring system

The subjects were monitored for 2 weeks before and after treatment with CGMS (Abbott, USA). The fluctuation index and its calculation formula were as follows:

#### Time in range (TIR^1^ and TIR^2^)

The percentage of time that blood glucose was in the range of 3.9–10.0 mmol/l during 24 h was TIR^1^, and the percentage of time between 0:00 and 6:00 am during the night that blood glucose was within the range of 3.9–10.0 mmol/l was TIR^2^.

#### Time below range (TBR^1^ and TBR^2^)

The percentage of time with blood glucose below 3.9 mmol/l in 24 h was TBR^1^, and the percentage of time with blood glucose below 3.9 mmol/l between 0:00 and 6:00 am during the night was TBR^2^.

#### Time above range (TAR^1^ and TAR^2^)

The percentage of time with blood glucose above 10 mmol/l in the 24 h was TAR^1^ and the percentage of time between 0:00 and 6:00 am during the night that blood glucose was higher than that of 10 mmol/l was TAR^2^.

#### Mean blood glucose (MBG^1^) and standard deviation (SD^1^)

The average of blood glucose during 24 h and its SD.

#### Mean ambulatory glucose excursions (MAGE^1^)

The average of all glucose excursions.

#### Absolute mean of daily differences (MODD)

The mean absolute deviation of matched values between two consecutive 24 h.

#### Mean blood glucose level (MBG^2^) and its standard deviation (SD^2^)

The average of blood glucose from 0 to 6 am during the night and its SD.

#### Mean ambulatory glucose excursions at night (MAGE^2^)

The average of all glucose excursions from 0 to 6 am during the night.

### Laboratory biochemical examination

The biochemical blood tests included postprandial blood glucose (PBG), fasting blood glucose (FBG), glycosylated hemoglobin (HbA1c), fasting insulin (FINS), uric acid (UA), serum creatinine (sCr), total cholesterol (TC), high-density lipoprotein cholesterol (HDL-C), triglycerides (TG), and low-density lipoprotein cholesterol (LDL-C). All blood tests were performed at Peking University International Hospital. HbA1c was quantified using high-performance liquid chromatography. PBG, UA, sCr, FBG, TC, TG, HDL-C, and LDL-C were measured using enzyme-based techniques (HPLC). Urinary microalbuminuria, urinary creatinine, and UACR values were calculated using the immunoturbidimetric method from our laboratory. The formula FINS (mIU/L) FBG (mmol/L)/22.5 was used to calculate the homeostasis model assessment index of insulin resistance (HOMA-IR).

### CPAP therapy

After CGMS monitoring, 30 subjects were assigned to the intervention group. The intervention group was treated with a fully automatic single level continuous positive pressure S9 ventilator (RESMED, USA). We adjusted the pressure level of CPAP according to the AHI of the patients, so that the patients could receive CPAP treatment without discomfort. The subjects were treated for at least 6 weeks and at least 4 h a night. All subjects were given lifestyle guidance, and the glucose-lowering guidance before and after treatment was not changed.

### Statistical analysis

Statistical analysis was carried out using the SPSS Version 21.0 software (IBM, Chicago, IL, USA) and used one-way analysis of variance (ANOVA) to compare the intervention group and control group. The *χ*^2^ test count data was used to compare count data among the groups and paired *t*-test was used to compare the data before and after treatment. The value of *P* less than 0.05 is considered to be statistically significant.

## Results

### CPAP therapy results

There were 30 patients (16 men) in the intervention group, with 17 having mild to moderate OSA and 13 having severe OSA, with an average age of 52.0 ± 11.2 years. In the control group of 30 patients (19 men), the average age was 54. 8 ± 9.0 years. CPAP treatment for the intervention group was well tolerated with average duration on therapy 55.2 ± 4.3 days and average daily wearing time at 8. 3 ± 2.8 h.

### Comparison of laboratory indexes between intervention group and control group before and after treatment

**Fig. 1:**
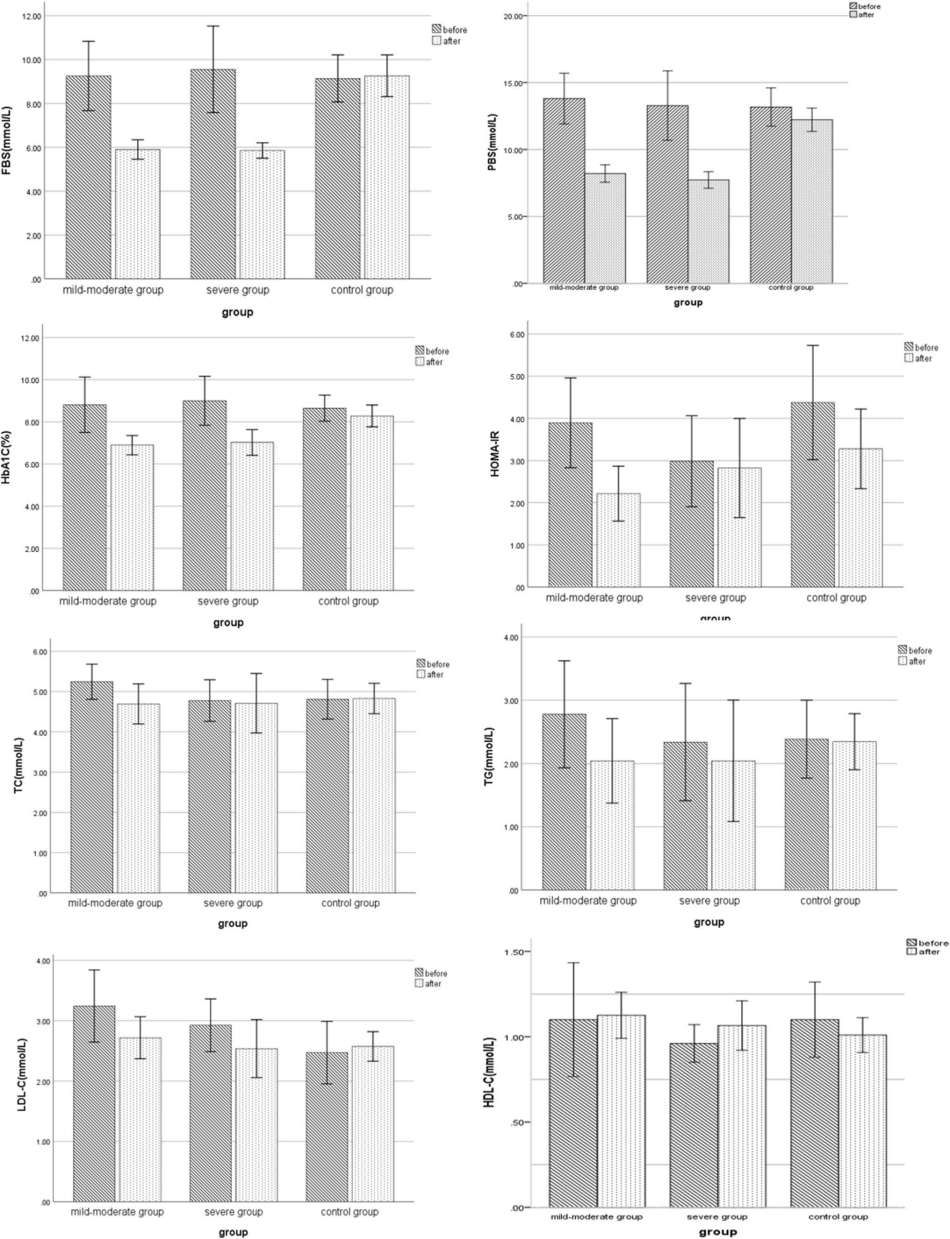
Comparison of laboratory indexes between intervention group and control group before and after treatment. There are no significant differences in LDL-C, TG, HDL-C, and TC, between the three groups. The level of HbA1c, PBG, and FBG reduced significantly compared with before in the intervention group (*P* < 0.05). In the control group, however, there were no significant differences in HbA1c, FBG, or PBG when compared to baseline (*P* > 0.05)

For the following attributes, there were no significant differences between the groups: gender, age, diabetic duration, WHR, FBG, PBG, BMI, HbA1c, TC, LDL-C, TG, UA, sCr, HOMA-IR, UACR, and HDL-C. After CPAP treatment, there were no significant differences in LDL-C, TG, HDL-C, TC, UA, UACR, sCr, and HOMA-IR between the groups. The level of HbA1c, PBG, and FBG decreased significantly compared with before CPAP in the intervention group (*P* < 0.05). In the control group, however, there were no significant differences in HbA1c, FBG, or PBG when compared to baseline measurements (*P* > 0.05) (shown in Table 1 and Fig. 1).

**Table 1:** Comparison of laboratory indexes between intervention group and control group before and after treatment

Index	Control group (*n* = 30)			Mild-moderate group (*n* = 17)			Severe group (*n* = 13)			*F*(*X*^2^)	*p*
	Before	After	*p*	Before	After	*p*	Before	After	*p*		
Age (years)	54.8 ± 9.0	-	-	52.8 ± 10.3	-	-	51.1 ± 14.1	-	-	-	-
Sex (male%)	19(63%)	-	-	6(35%)	-	-	10(77%)	-	-	-	-
BMI (kg/m^2^)	30.9 ± 4.5	30.2 ± 4.4	0.06	30.2 ± 3.3	30.2 ± 3.34	0.57	30.3 ± 4.2	30.2 ± 4.3	0.23	1.78	0.18
WHR	0.99 ± 0.07	0.97 ± 0.32	0.34	0.99 ± 0.06	1.00 ± 0.21	0.35	0.98 ± 0.09	0.97 ± 0.01	0.46	0.31	0.78
Duration (years)	6.7 ± 6.8	-		8.6 ± 6.5	-		4.0 ± 5.1	-		-	-
AHI	34.5 ± 20.4	-		16.0 ± 7.3	3.5 ± 2.2	< 0.05	44.4 ± 14.2	4.5 ± 2.6	< 0.05	1.25	0.27
SBP (mmHg)	136.6 ± 7.6	130.3 ± 8.2	0.31	129.2 ± 3.9	130.3 ± 4.1	0.23	129.7 ± 3.5	127.3 ± 7.3	0.56	1.02	0.42
DBP (mmHg)	82.7 ± 13.9	83.0 ± 20.3	0.67	89.7 ± 7.4	86.4 ± 11.0	0.24	87.3 ± 6.5	80.4 ± 8.6	0.43	2.09	0.29
FBG (mmol/L)	9.14 ± 2.87	9.26 ± 2.54	0.55	9.25 ± 3.07	5.90 ± 0.86	< 0.05	9.55 ± 3.26	5.85 ± 0.58	< 0.05	23.98	< 0.05
PBG (mmol/L)	13.20 ± 3.83	12.22 ± 2.32	0.18	13.81 ± 3.68	8.21 ± 1.26	< 0.05	13.28 ± 4.29	7.72 ± 1.03	< 0.05	39.29	< 0.05
HbA1c (%)	8.65 ± 1.66	8.28 ± 1.39	0.09	8.81 ± 2.55	6.89 ± 0.89	< 0.05	9.00 ± 1.92	7.02 ± 1.01	< 0.05	9.41	< 0.05
TC (mmol/L)	4.77 ± 1.32	4.83 ± 0.99	0.79	5.24 ± 0.85	4.69 ± 0.97	0.10	4.78 ± 0.85	4.71 ± 1.22	0.86	0.11	0.89
TG (mmol/L)	2.39 ± 1.68	2.34 ± 1.16	0.91	2.78 ± 1.64	2.04 ± 1.30	0.21	2.34 ± 1.53	2.04 ± 1.59	0.67	0.40	0.67
LDL-C (mmol/L)	2.51 ± 1.39	2.58 ± 0.64	0.81	3.24 ± 1.16	2.72 ± 0.68	0.15	2.93 ± 0.72	2.54 ± 0.79	0.14	0.32	0.73
HDL-C (mmol/L)	1.08 ± 0.45	0.99 ± 0.21	0.32	1.10 ± 0.65	1.13 ± 0.26	0.90	0.96 ± 0.18	1.07 ± 0.24	0.17	0.98	0.38
UA (umol/L)	399.9 ± 87.3	350.6 ± 81.3	0.06	361.0 ± 131.5	315.6 ± 81.6	0.34	334.4 ± 84.0	320.2 ± 85.1	0.65	1.29	0.28
sCr (mmol/L)	80.6 ± 30.5	69.4 ± 17.2	0.10	66.2 ± 16.2	68.9 ± 16.3	0.62	69.9 ± 18.0	72.7 ± 24.5	0.77	0.39	0.68
UACR	31.4 ± 32.1	8.9 ± 5.7	< 0.05	32.3 ± 67.9	9.9 ± 5.8	0.24	34.0 ± 69.0	24.7 ± 55.4	0.06	0.56	0.58
HOMA-IR	4.28 ± 3.70	3.28 ± 2.53	0.22	3.89 ± 2.07	2.21 ± 1.27	< 0.05	2.98 ± 1.78	2.82 ± 1.94	0.78	1.37	0.26

*BMI* body mass index; *WHR* waist-to-hip ratio; *SBP* systolic blood pressure; *DBP* diastolic blood pressure; *FBG* fasting blood glucose; *HbA1c* glycosylated hemoglobin; *sCr* serum creatinine; *UA* uric acid; *TC* total cholesterol; *TG* triglycerides; *LDL-C* low-density lipoprotein cholesterol; *HDL-C* high-density lipoprotein cholesterol; *PBG* postprandial blood glucose; *UACR* urinary microalbumin/creatinine ratio; *HOMA-IR* homeostasis model assessment index of insulin resistance

### Comparison of blood glucose fluctuation indexes between intervention group and control group before and after treatment

**Fig. 2:**
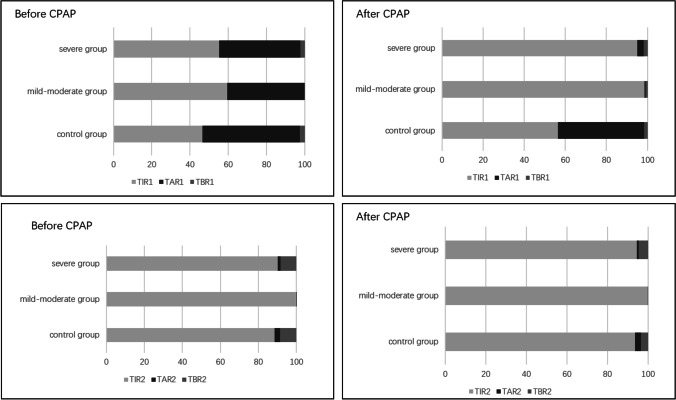
Comparison of blood glucose fluctuation indexes between intervention group and control group before and after treatment. The levels of TIR^1^, TAR^1^, TBR^1^, TIR^2^, TAR^2^, and TBR^2^ did not differ significantly between the control group and the intervention group before treatment (*P* > 0.05). The level of TIR^1^ and TAR^1^ were lower than those before treatment in the intervention group (*P* < 0.05)

The levels of MBG^1^, MAGE^1^, MODD, SD^1^, MBG^2^, SD^2^, MAGE^2^, TIR^1^, TAR^1^, TBR^1^, TIR^2^, TAR^2^, and TBR^2^ did not differ significantly between the control group and the intervention group before treatment (*P* > 0.05). MBG1, MAGE1, MODD, and SD1 in the intervention group were considerably lower after CPAP treatment than before treatment (*P* < 0.05), and TIR^1^ and TAR^1^ were lower than those before treatment (*P* < 0.05). Furthermore, the findings revealed substantial alterations in MBG2, SD2, and MAGE2 before and after therapy (*P* > 0.05). However, the levels of MAGE^1^, MODD, SD^1^, MBG^2^, SD^2^, and MAGE^2^ did not change significantly in the control group (shown in Table 2 and Fig. 2).

**Table 2:** Comparison of blood glucose fluctuation indexes between intervention group and control group before and after treatment

Index	Control group (n = 30)			Mild-moderate group (*n* = 17)			Severe group (*n* = 13)			*F*(*X*^2^)	*p*
	Before	After	*p*	Before	After	*p*	Before	After	*p*		
MBG^1^ (mmol/L)	8.50 ± 2.13	7.90 ± 1.23	0.12	8.02 ± 1.58	6.66 ± 0.93	< 0.05	8.06 ± 2.27	6.17 ± 1.41	< 0.05	11.68	< 0.05
SD^1^ (mmol/L)	2.22 ± 1.02	2.29 ± 1.04	0.44	1.75 ± 0.88	1.08 ± 0.31	< 0.05	1.82 ± 0.86	0.78 ± 0.43	< 0.05	22.25	< 0.05
MAGE^1^ (mmol/L)	4.71 ± 2.09	4.29 ± 1.03	0.28	3.75 ± 1.36	2.44 ± 0.80	< 0.05	3.82 ± 1.43	2.08 ± 1.40	< 0.05	25.74	< 0.05
MODD (mmol/L)	2.16 ± 0.97	2.09 ± 0.85	0.58	1.60 ± 0.94	1.14 ± 0.40	< 0.05	1.97 ± 1.22	0.95 ± 0.59	< 0.05	15.92	< 0.05
MBG^2^ (mmol/L)	6.69 ± 1.91	6.08 ± 1.19	0.12	6.65 ± 1.15	5.94 ± 0.96	< 0.05	6.72 ± 2.31	5.87 ± 1.41	0.12	0.01	0.99
SD^2^ (mmol/L)	0.78 ± 0.50	0.76 ± 0.43	0.78	0.55 ± 0.36	0.35 ± 0.16	< 0.05	0.89 ± 0.66	0.48 ± 0.40	< 0.05	1.49	0.24
MAGE^2^ (mmol/L)	1.90 ± 1.30	1.65 ± 1.34	0.17	1.52 ± 1.29	0.91 ± 0.73	< 0.05	2.19 ± 1.51	1.22 ± 1.43	< 0.05	2.09	0.13
TIR^1^ (%)	46.64 ± 28.72	56.35 ± 29.04	0.10	59.47 ± 25.62	98.46 ± 3.02	< 0.05	55.22 ± 30.92	94.96 ± 12.16	< 0.05	27.55	< 0.05
TAR^1^ (%)	50.92 ± 30.06	41.92 ± 30.06	0.09	40.47 ± 25.70	0.42 ± 1.29	< 0.05	42.56 ± 31.22	3.17 ± 11.13	< 0.05	30.08	< 0.05
TBR^1^ (%)	2.43 ± 6.29	1.73 ± 5.97	0.68	0.06 ± 0.24	1.12 ± 2.91	0.16	2.22 ± 5.35	1.87 ± 5.77	0.89	0.18	0.83
TIR^2^ (%)	88.48 ± 23.90	93.72 ± 21.63	0.31	99.95 ± 0.19	99.86 ± 0.59	0.33	90.19 ± 32.46	94.42 ± 14.19	0.31	2.00	0.15
TAR^2^ (%)	3.00 ± 13.32	2.86 ± 12.05	0.65	0.05 ± 0.61	0	0.33	1.54 ± 5.54	1.08 ± 3.88	0.34	0.62	0.54
TBR^2^ (%)	8.52 ± 21.14	3.42 ± 11.92	0.28	0	0.14 ± 0.59	0.23	8.27 ± 20.55	4.62 ± 14.11	0.63	0.82	0.45

*MBG*^*1*^ average blood glucose level; *SD*^*1*^ standard deviation of average blood glucose level; *MAGE*^*1*^ the average fluctuation of blood glucose in the day; *MODD* mean absolute difference of daytime blood glucose; *MBG*^*2*^ mean blood glucose level at night; *SD*^*2*^ standard deviation of average blood glucose level at night; *MAGE*^*2*^ the mean fluctuation of blood glucose at night; *TIR*^*1*^ and *TIR*^*2*^ the time percentage of blood glucose in the range of 3.9–10.0 mmol/l during 24 h was TIR^1^, and the time percentage between 0:00 and 6:00 in the night in the range of 3.9–10.0 mmol/l during night was TIR^2^; *TBR*^*1*^ and *TBR*^*2*^ the time percentage of blood glucose below 3.9 mmol/l in 24 h was TBR^1^, and the time percentage between 0:00 and 6:00 in the night in the range of 3.9–10.0 mmol/l was TBR^2^; *TAR*^*1*^ and *TAR*^*2*^:the time percentage of blood glucose in the range above 10 mmol/l was TAR^1^, and the time percentage between 0:00 and 6:00 in the night in the range of above 10 mmol/l was TAR^2^

## Discussion

In our study, after CPAP treatment, the glucose metabolism in the intervention group including FBG, PBG, and HbA1c improved significantly compared to the control group. At the same time, the BMI of the intervention group did not change significantly with CPAP treatment (*P* > 0.05). CPAP improved blood glucose levels in patients with T2DM regardless of body weight, and blood glucose levels in the intervention group were considerably lower than those in the control group. This study found that insulin resistance in patients with mild to moderate OSA decreased significantly, which is similar to most current research results.

It has previously been controversial whether or not CPAP treatment can effectively improve insulin resistance, decrease HbA1c, FBG, and PBG, and improve blood glucose metabolism in patients with OSA and T2DM [10, 11]. Two meta-analyses have shown that CPAP may not improve glycemic control [12, 13]. Also, there is limited evidence that CPAP therapy can increase a patient’s glucose time in range.

### The mechanism of impaired glucose stability induced by OSA

At present, the mechanisms of impaired glucose stability induced by OSA in diabetes include the following:

#### Activation of the sympathetic nervous system

OSA causes sleep fragmentation and sympathetic adrenal axis excitability, which reduces tissue sensitivity to insulin, insulin secretion, and hepatic glucose breakdown, aggravating insulin resistance [14].

#### Direct effects of hypoxia

OSA-induced intermittent hypoxia and reoxygenation can result in intermittent tissue hypoxia and reoxygenation, which differs from chronic hypoxia. The continuous fluctuations in oxygen saturation contribute to the formation of reactive nitrogen and oxygen, increase oxidative stress, and activate the redox-sensitive cell signal transduction pathway in inflammation [15, 16]. Hypoxia can cause stress, mitochondrial damage, inflammation, and β cell apoptosis [17].

#### Sleep fragmentation

Patients with OSA have sleep fragmentation and frequent awakening, and sudden sleep fragmentation can reduce insulin sensitivity. A recent animal study [18] found that during natural sleep, insulin sensitivity of visceral adipocytes exposed to sleep interruption was reduced.

#### Systemic inflammatory response

Sleep fluctuation can cause systemic inflammation. Tumor necrosis factor (TNF-α) and the level of interleukin-6 (IL-6) is increased, and the phosphorylation of insulin receptor and insulin receptor substrate is inhibited. These inflammatory factors can affect glucose metabolism by increasing the hormone level against insulin and inhibiting the intake of glucose by muscle tissue and fat [19]. The level of inflammatory factors in the body is related to the fluctuation of blood glucose. In diabetes, good blood glucose control and reasonably constant blood glucose levels are advantageous in reducing the inflammatory response in vivo.

The damage of tissue and organs caused by glucose metabolism disorder is not only determined by the degree of blood glucose increase but also closely related to the fluctuation range of blood glucose. In diabetes patients with similar blood glucose or HbA1c, the magnitude of blood glucose fluctuation is quite variable. Similarly, blood glucose fluctuations have been shown to be a significant component in the pathogenesis of diabetes macrovascular and microvascular problems. Compared with chronic persistent hyperglycemia, blood glucose fluctuation has a more serious effect on the occurrence and development of diabetes complications [20]. The fluctuation of intraday blood glucose monitored by CGMS is closely related to vascular endothelial dysfunction, vascular stress, diabetes microangiopathy [21–24], and severity of T2DM. The current study not only investigated how blood glucose levels improved in patients with T2DM and OSA but also evaluated how blood glucose levels fluctuated after CPAP treatment. The results showed that, after treatment, the blood glucose fluctuation indexes of the intervention group, including MAGE, SD, and MODD, were much lower, and the differences were statistically significant compared to the control group. The results showed that the levels of MAGE and SD decreased significantly in the night compared with the control group. Similarly, prior studies [25, 26] have demonstrated that CPAP treatment may significantly improve the blood glucose fluctuation and blood glucose level of nocturnal interstitial fluid in patients with T2DM and OSA.

TIR is currently being utilized as a novel blood glucose evaluation index for assessing the variability of blood glucose in patients with diabetes. It has been shown that the shorter the TIR, the higher the incidence of microalbuminuria and retinopathy in diabetes [22]. In a retrospective study of 9028 critically ill patients with or without diabetes, the lower the TIR, the greater the chance of death [27]. The results in our study showed that the TIR and TAR levels of the intervention group were dramatically improved with CPAP treatment.

This study has limitations. First, the average age of patients in our study was too elderly to reflect all patients with T2DM. Second, the follow-up period was limited to 3 months due to time constraints, and it was not known if the improvements in blood glucose levels and their fluctuations would be durable. Third, the study sample size of 60 participants is relatively small, limiting the generalizability of the findings.

## Conclusions

CPAP improves blood glucose levels and reduces fluctuation of blood glucose levels in patients with T2DM and OSA. It is an effective way to improve abnormal glucose metabolism in patients with T2DM and OSA, beyond lifestyle intervention and drug therapy.

## Data Availability

The data used to support the findings of this study are available from the corresponding author and first author upon request.
